# 

**Published:** 1884-01

**Authors:** 


					﻿SURGGEKlS'OEJS ]
Voli XIX.
January, 1884.
No. 4.
The
1STDURY.
Crf 5L0® MB
EDITOR-,
amiUMY.
				

